# Influence of an exercise program on cardiac remodeling and functional capacity in patients with stroke (CRONuS trial): study protocol for a randomized controlled trial

**DOI:** 10.1186/s13063-019-3328-1

**Published:** 2019-05-28

**Authors:** Josiela Cristina da Silva Rodrigues, Gustavo José Luvizutto, Rafael Dalle Molle da Costa, Robson Aparecido Prudente, Taís Regina da Silva, Juli Thomaz de Souza, Caroline Ferreira da Silva Mazeto Pupo da Silveira, Daniele Andreza Antonelli Rossi, Fernanda Cristina Winckler, Gabriel Pinheiro Modolo, Tainá Fabri Carneiro Valadão, Letícia Cláudia de Oliveira Antunes, Luis Cuadrado Martin, Rodrigo Bazan, Silméia Garcia Zanati Bazan

**Affiliations:** 1 Department of Rehabilitation, Botucatu Medical School (UNESP), São Paulo State University, São Paulo, Brazil; 2 Department of Applied Physiotherapy, Triangulo Mineiro Federal University, Uberaba, Brazil; 3 Department of Neurology, Psychology and Psychiatry, Botucatu Medical School (UNESP), São Paulo State University, São Paulo, Brazil; 4 Department of Internal Medicine, Botucatu Medical School (UNESP), São Paulo State University, District of Rubião Junior, Botucatu, SP 18618-687 Brazil

**Keywords:** Stroke, Physical exercise, Cardiovascular rehabilitation, Echocardiography, Functional capacity

## Abstract

**Background:**

Cardiovascular rehabilitation is one of the treatment options for post-stroke individuals in order to improve functional independence in activities of daily life and reduce energy expenditure. The aim of this trial is to evaluate the effect of an exercise program on the echocardiographic variables, functional capacity, inflammatory response, neurological status, nutritional status, cardiologic evaluation, and quality of life of patients after stroke.

**Methods/design:**

This is a randomized controlled trial including patients with ischemic stroke in the chronic phase. The patients will be evaluated at the beginning of the study and after 16 weeks. This will include clinical and physical evaluation, 6-min walk test, neurological assessment, nutritional assessment, ambulatory blood pressure monitoring, transthoracic echocardiography, and assessment of the quality of life. The sample size has been determined as 40 patients, who will be divided into two groups: control group (CG; *n* = 20) and intervention group (IG; n = 20). The CG will undergo conventional physiotherapy for 45 min, three times a week, up to 16 weeks, while the IG will be put on a cardiovascular rehabilitation program consisting of heating, aerobic exercise, and muscle strengthening for 45 min, three times a week, for 16 weeks. The primary endpoint will be functional capacity following a 6-min walk test (delta maxVO_2_) and morphofunctional echocardiographic variables (indexed left ventricular mass) before and after the intervention.

**Discussion:**

We expect to observe an improvement in cardiac structural and functional abnormalities in the IG, on echocardiography and biochemical examination, and that the improvement of these parameters after cardiovascular rehabilitation will have a favorable impact on the functional capacity and quality of life of patients after stroke.

**Trial registration:**

REBEC, RBR-4wk4b3. Registered on 19 September 2016.

**Electronic supplementary material:**

The online version of this article (10.1186/s13063-019-3328-1) contains supplementary material, which is available to authorized users.

## Background

Worldwide, stroke is a leading cause of morbidity and mortality, affecting around 200,000 individuals annually. In addition, it is a major cause of chronic disability in adults, with major impacts on the health and quality of life of the affected population [1, 2].

Stroke is defined as an episode of sudden neurological dysfunction of ischemic or hemorrhagic origin, with persistent clinical symptoms over an hour and evidence of demonstrable lesion on imaging tests [3]. Pathologically, stroke can be defined as brain cell death resulting from a prolonged ischemic event [4].

The incidence of stroke is greater in men aged 45–85 years and in women over 85 years. The major risk factors for ischemic stroke are increased blood pressure, diabetes mellitus (DM), cardiac arrhythmias, dyslipidemia, smoking, physical inactivity, genetic or family history of stroke, chronic kidney disease, and sleep apnea syndrome [5]. The etiology of hemorrhagic stroke can be primary (hypertension or amyloid angiopathy) or secondary to vascular malformations, such as aneurysm, cavernous angioma, venous angioma, venous sinus thrombosis, vasculopathies, and central nervous system tumors, among others [6].

After a stroke, individuals require long periods of rehabilitation to increase their functional capacity and to minimize the sequelae resulting from brain injury [7, 8]. In the chronic phase of stroke, 62% of patients need assistance in activities of daily living (ADL), and less than half of patients are able to perform independent walking, which is the most disabling outcome of the disease, decreasing their overall physical capacity [9–11].

Inactivity through reduced mobility and low levels of aerobic capacity after stroke result in several dysfunctions, such as reduction in cardiorespiratory fitness, loss of 20% of the cross-sectional area of the muscle, and an increase of about 25% of intramuscular fat, leading to osteoporosis. There is also the circulatory involvement of the lower extremities, as well as psychological effects such as apathy and depression [8–12]. In association with other comorbidities such as DM, hypertension, and increased body mass index, the risk of associated cardiovascular events may increase in addition to recurrence of stroke [13].

In conventional rehabilitation after stroke, the goal is to achieve maximum functional recovery [14, 15]. However, approximately 75% of individuals have associated heart disease after experiencing a stroke, thereby reducing their functional capacity to perform ADL [16–18]. Immobility after stroke results in resistance to physical exercise, including ADL [19, 20].

Several studies have reported that the maximum oxygen capacity (VO_2max_), defined as the critical (peak) oxygen capacity during aerobic activity, is reduced to 10–17 ml/kg/ min in the first 30 days after stroke, and does not reach values > 20 ml/kg/min after 6 months of the event [21–26]. VO_2max_ values after stroke decrease by 25–45% compared to healthy individuals in the same age group, which may interfere with the rehabilitation process and functional prognosis in the long term [27, 28].

Therefore, cardiorespiratory rehabilitation aims to provide greater functional independence during ADL with lower energy expenditure [28]. Several authors have reported that conventional rehabilitation is effective in improving the functional independence of individuals after stroke, but cardiorespiratory rehabilitation can restore aerobic capacity, reduce energy expenditure to perform ADL, and reduce the occurrence of cardiac events and recurrence of stroke [14, 15, 19, 20, 26–28]. However, the type, frequency, and appropriate method to achieve these goals during cardiorespiratory rehabilitation remain unclear in the literature. Hence, this study has clinical importance.

The main aim of this study is to evaluate the effect of a cardiovascular rehabilitation program on morphofunctional echocardiographic variables and functional capacity in patients with chronic ischemic stroke. In addition, we will assess the effects of cardiovascular rehabilitation on the inflammatory response, neurological status, nutritional status, cardiologic evaluation, and quality of life. We also aim to evaluate the association between clinical variables (age, anthropometric data, mean systolic blood pressure, 24-h diastolic blood pressure, magnitude of the nocturnal decrease in systolic blood pressure, diastolic and pulse pressure, use of antihypertensive medications) and echocardiographic variables and VO_2max_.

## Methods/design

### Design

**Fig. 1:**
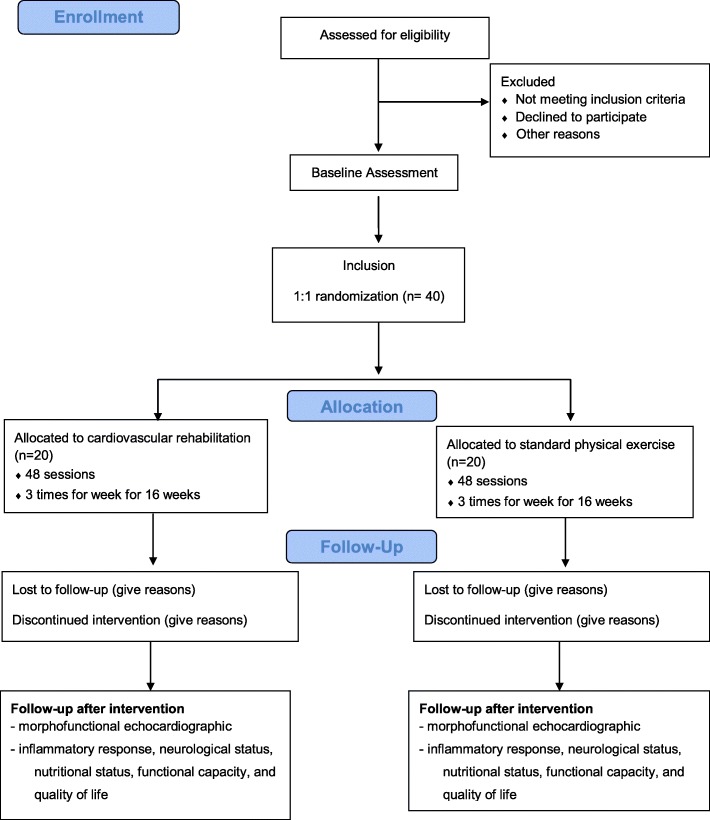
Study flow diagram

This single-center, randomized, single-blind, parallel group study of 40 patients with stroke will be conducted in accordance with the Consolidated Standards of Reporting Trials (CONSORT) 2010. A flow diagram of the study is presented in Fig. 1.

### Patient population

Participants were recruited from the Cardiac RemOdeliNg and functional capacity in patients with Stroke (CRONuS) trial, a single-center, randomized, single-blinded study of cardiac rehabilitation in 40 individuals with stroke at UNESP – University Estadual Paulista, Botucatu Medical School, Brazil.

### Inclusion criteria

Participants older than 18 years of either sex, with a history of ischemic stroke, modified Rankin scale (mRS) ≤ 2, and currently in the chronic phase of stroke (6 to 12 months after onset of symptoms), but clinically stable to participate in the study after discharge from hospital [28].

### Exclusion criteria

Patients with hemorrhagic stroke, mRS > 2, uncontrolled hypertension (systolic blood pressure ≥ 150 mmHg), history of angina in the last 3 months, uncontrolled epilepsy, persistent or permanent atrial fibrillation and/or using a pacemaker, presence of valvulopathies such as mitral and/or aortic stenosis, presence of grade IV heart failure, stage D chronic obstructive pulmonary disease, and life expectancy < 6 months.

## Randomization and blinding

The concealed randomization schedule will be established using a computer-generated random number sequence and maintained by an offsite investigator who will not be involved with the enrolment nor the assessment of study participants. A second research assistant will sequentially open consecutively numbered, randomly ordered, opaque envelopes containing the group allocation (in a 1:1 ratio) after baseline assessment. The patients will be evaluated before the beginning of the first session and a week after the last session by a blinded investigator.

## Intervention

Frequency, duration, intensity, and supervision standards will be established for the implementation and maintenance of the cardiovascular rehabilitation program, which proposes approximately 45 min of exercises, three times a week, for 16 weeks (48 sessions), consisting of the following three phases [28–30]:

- Heating: light walk for a duration of 5 to 10 min.
- Aerobic exercises: 30 min of aerobic exercise on the treadmill without programmed increase in speed, compatible with walking ability of the patient, and achieving approximately 40 to 80% of maximum heart rate reserve. It is calculated by the Karvonen formula for target heart rate where Target heart rate = [% intensity × (Maximum HR − Resting HR)] + Resting HR; it can be readjusted according to the sessions and is progressive. This phase will take place under professional supervision.
- Muscle cooling: static stretching of the hamstrings, quadriceps, sural triceps, and hip adductors for 5–10 min.

The protocol will include physiologic monitoring of blood pressure, heart rate, respiratory rate, peripheral oxygen saturation, and perceived exertion (modified BORG scale). The individual will also be instructed to stop the activity if any discomfort is felt.

Patients in the control group will receive conventional outpatient physiotherapeutic care at the Clinical Hospital, UNESP—University Estadual Paulista, Botucatu Medical School, Brazil. No changes will be made to the standard hospital protocol in terms of the content, time, or duration of the session. The protocol will be based on physiotherapeutic intervention guided by the National Institute for Health and Care Excellence (2013), with exercises consisting of 10 min of stretching exercises for the lower limbs, 15 min of strengthening exercises with emphasis on core, knee extensors and flexors, plantar flexors and dorsiflexors, and 20 min of gait training with obstacles and variabile speed. Patients will wear orthoses for gait training physical therapy three times a week for 16 weeks, for 48 sessions in total.

### Primary outcome measures

The primary endpoint will be measured as functional capacity following a 6-min walk test (delta maxVO_2_) and morphofunctional echocardiographic variables (indexed left ventricular mass) before and after the intervention.

### Secondary outcome measures

The secondary endpoints will be inflammatory responses, neurological status, nutritional status, cardiologic evaluation, and quality of life.

### Adverse effects

Adverse effects, frequency, duration, exercise intensity, and/or other relevant events will be recorded every week.

## Procedures (template)

**Fig. 2:**
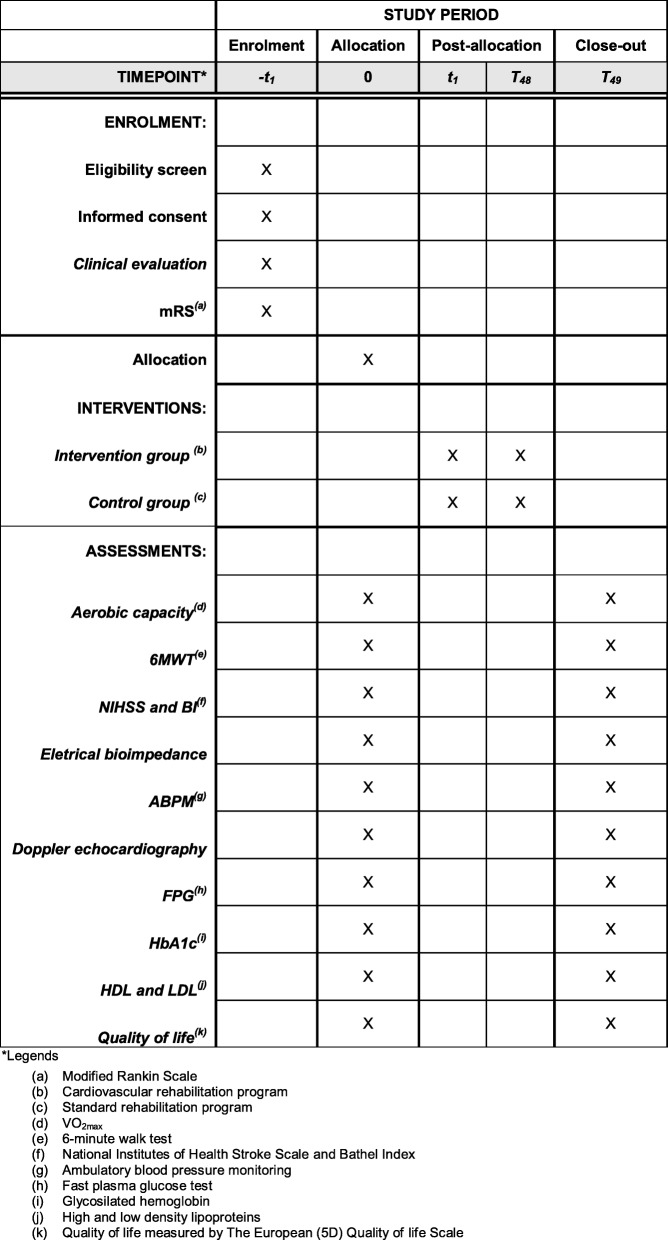
Template of recommended content for the schedule of enrolment, interventions, and assessments. *6MWT* 6-min walk test, *ABPM* ambulatory blood pressure monitoring, *BI* Barthel Index, *LDL* low-density lipoprotein, *HDL* high-density lipoprotein, *FPG* Fast plasma glucose test, to evaluate the glycemic control

Individuals diagnosed with ischemic stroke will be sent to the rehabilitation center after discharge from the hospital. All individuals with ischemic stroke confirmed by a computed tomography (CT) or magnetic resonance imaging (MRI) scan will be invited to participate in the study. The patient, a family member, or guardian must sign an informed consent form. The individuals will then be evaluated for their clinical and neurological status. Individuals will be assessed using the National Institutes of Health Stroke Scale (NIHSS), the Barthel Index of ADL, and the mRS. The aerobic capacity will be evaluated by VO_2max_, functional capacity will be assessed by a 6-min walk test, nutritional status by electric bioimpedance, cardiologic examination by ecocardiography, and laboratory exams and the quality of life will be based on the European Quality of Life Scale. The tests will be performed by an investigator who is blinded to the treatment that the patient received before the first session and a week after the last session. After the clinical screening, they will be randomized into the following two groups: 1) control group (CG; 20 patients); 2) intervention group (IG; 20 patients) (Fig. 2).

## Data monitoring

Patients of both groups, CG and IG, will be evaluated initially and after 16 weeks of research, using the following procedures.

### Clinical evaluation

The clinical evaluation will include anamnesis and a general physical examination, recording demographics, morbidities, and medications in use.

### Physical assessment

The physical evaluation will consist of anthropometric measures and evaluation of the peripheral muscular strength.

### Evaluation of functional capacity

#### Aerobic capacity

A maximum stress test will be used to obtain VO_2max_, peak work rate, and anaerobic ventilatory threshold. The heart rate and resting blood pressure will be measured before and after the exercise. The stress test will be based on a modified Bruce protocol that uses a time-dependent slope [31]. The protocol will be based on the study by Tang et al. [32], which used 5, 10, or 15-watt work rates for a total test duration of between 8 and 10 min.

#### Six-minute walk test

The 6-min walk test establishes functional fitness of gait through performance and physical resistance. The test measures the maximum distance reached along a course of 30 m in 6 min. The expected distance to be reached by the patient will be calculated based on age, sex, weight, and height [33, 34]. Equipment required for the test include a stopwatch, cones for circuit delimitation, sphygmomanometer, stethoscope, and pulse oximeter. According to the protocol proposed by the American Thoracic Society guidelines, the evaluator should not walk with the patient, but may walk behind the patient if necessary to carry the oxygen source or in case the patient loses balance. During the test, encouraging (standardized) phrases should be used over time. Vital signs are checked at the beginning and end of the test [35].

### Neurological and functional evaluation

#### National Institutes of Health Stroke Scale

The NIHSS is an assessment to quantify the neurological deficits as well as the severity of the stroke. The assessment can be performed by both medical staff and health professionals with proven training and certification [1].

#### Modified Rankin Scale

The mRS is to assess the degree of independence and determine if patients can care for themselves during ADL [1]. The scale ranges from 0 to 6, and the greater the score, the lower the functional capacity.

#### Barthel Index

The Barthel Index will be used to measure the functional independence of individuals in 10 ADL, and the score varies from 0 to 100. A score above 95 indicates that an individual is independent in all ADL [1].

### Nutritional assessment

#### Electrical bioimpedance

Electrical bioimpedance is a non-invasive, practical, and reproducible method for evaluation of the body composition and distribution of intra- and extracellular fluids of a patient [36, 37]. Based on the principle that different body tissues offer different resistance levels to the passage of an electric current, electrodes are placed at the tips of the thumbs, middle fingers, and heels for passing a low intensity electrical current. The amount of resistance offered helps in assessing tissue differentiation and distribution of body fat [37]. It also provides data on body mass index, which is an anthropometric indicator to establish nutritional status and measure abdominal circumference as an indicator of chronic diseases.

### Cardiological evaluation

#### Ambulatory blood pressure monitoring

Ambulatory blood pressure monitoring is a diagnostic method that monitors 24-h changes in blood pressure, during both wakefulness and sleep, and its effect on the patient. It allows a better understanding of systemic arterial hypertension for diagnosis, prognosis, and appropriate treatment [38]. The device will be programmed for readings at 20-min intervals during wakefulness and 30 min during sleep, with a minimum of 14 readings when awake and seven during sleep. The period of vigilance will be between 7 am and 10 pm. At least two test measures will be performed before the patient is discharged. The readings will be considered valid if there is a minimum of 24 h of recording and a minimum number of valid measures per hour (three during the waking hours and two during sleep). The following parameters will be analyzed: systolic mean arterial pressure, diastolic and 24 h pulse pressures (wakefulness and sleep), and magnitude of the nocturnal decrease in systolic, diastolic, and pulse pressures [39].

#### Doppler echocardiographic evaluation

The Doppler echocardiographic evaluations will be performed by a single examiner using a Vivid S6 (General Electric Medical Systems, Israel) with multi-frequency ultrasonic transducer 2.0–3.5 MHz. During the procedure, patients will remain in a left lateral decubitus position, with the left upper limb slightly flexed under the head. An electrocardiographic shunt will be continuously monitored.

The images will be obtained and analyzed following the recommendations of the American Society of Echocardiography [40, 41].

##### Morphometric variables

- Maximum left atrium diameter (LA, cm)
- Left atrial volume (LAV, mL), obtained by the Simpson method in two longitudinal planes, four and two chambers
- Left ventricular diastolic and systolic diameters of the left ventricle (LV, mm): LVDD and LVSD, respectively
- Interventricular septum diastolic thickness (IVSDT) and posterior wall diastolic thickness (PWDT) of the LV (mm): IVSDT and PWDT, respectively
- Relative thickness of the LV (LVRT) = (2 × PWDT)/LVDD
- Left ventricular mass (LVM, g) = 0.8 × {1.04 × [(IVSDT + PWDT + LVDD)^3^ − LVDD^3^]} + 0.6
- LVM index (LVMI, g/m^2.7^) = LVM/Height^2.7^ where LVMI is LV mass indexed to height.

##### Systolic function variables

- LV ejection fraction (LVEF), obtained by the Simpson biplane method
- Percentage of variation in ventricular diameter (%∆D) = [LVDD − LVSD)/LVDD] × 100
- Maximum systolic excursion velocity of the mitral annulus: S wave, obtained by spectral recording of the movement of mitral annulus, in its medial and lateral portions on tissue Doppler

##### Diastolic function variables

- LAV (mL), normalized to the body surface (LAVI, mL/m^2^)
- Maximum early ventricular filling velocity (E wave peak, cm/s): obtained by spectral Doppler recording of the transmitral diastolic flow
- Maximum late filling velocity during atrial contraction (A wave peak, cm/s): obtained by spectral Doppler recording of the transmitral diastolic flow
- E/A ratio
- Isovolumetric relaxation time of the LV (ms), corresponding to the period between the end of the ventricular ejection and the beginning of mitral transvalvular diastolic flow
- E-wave deceleration time (ms) corresponding to the time between the initial velocity peak of the mitral transvalvular flow and its extrapolation to the baseline
- Maximum excursion velocities of the mitral annulus, in the early ventricular filling phase (E’ medium, cm/s) and during the atrial contraction (A’ medium, cm /s), obtained by the spectral recording of tissue Doppler of movement of the ring mitral, in its medial and lateral portions
- E/E’ medium ratio

### Laboratory evaluation

A) Fasting plasma glucose test: to evaluate glycemic control. It reflects the lowest plasma glucose values of the day. Possible to establish comorbidities such as type 2 DM [42]
B) Glycosylated hemoglobin: allows the monitoring of glycemic control in diabetic patients since it provides information without large fluctuations on the retrospective index of plasma glucose and is directly related to the risk of complications in patients with type 1 and 2 DM [43]
C) High density lipoproteins and low density lipoproteins
D) Pro-inflammatory cytokines: evaluate levels of inflammatory response in the body, interleukins (IL-1β, IL-10, and IL-6), C-reactive protein, and tumor necrosis factors [44]

### Quality of life assessment

#### EuroQol

The European (5D) Quality of Life Scale (EuroQol) will be used to assess the impact of stroke on an individual’s quality of life through five parameters structured on mobility, personal care, usual activities, pain/discomfort, and anxiety/depression ranging from 0 to 10. The higher the score, the worse the perception of quality of life. At the end of the test, the patient should report their health on an ordinal scale of 0 to 100, and the closer to 0, the worse their condition, and the closer to 100, the better [45, 46].

## Sample size estimation

The sample size was calculated to match primary outcome with sample size estimation.

According to Framingham data [47], an increase of 20 g/m^2.7^ in left ventricular mass index presents a hazard ratio to hard end points of about 1.5; in this study the standard deviation was 30 g/m^2.7^. To detect these clinically significant differences between groups, with a fixed alpha error of 0.05 and beta error of 0.20, 37 patients per group will be needed.

According to the cohort of Presher et al. [48], an increase of 66 m in the 6-min walking test discriminates survivors from non- survivors among heart failure patients; in that study the standard deviation was 90 m. To detect these clinically significant differences between groups, with a fixed alpha error of 0.05 and beta error of 0.20, 31 patients per group will be needed.

Therefore, we will study 40 patients per group to detect these differences properly.

## Statistical analysis

Continuous variables will be expressed as means and standard deviations or medians and interquartile ranges. The associations between clinical and echocardiographic variables will be evaluated by Student’s *t*-test for the variables with normal distribution, or Mann-Whitney test with non-normal distribution for comparison between groups. Comparisons between the values before and after the intervention will be made using the paired *t*-test. The associations between the continuous variables, in the same group and before and after the intervention, will be evaluated by means of repeated measures ANOVA correlation test. Data will be analyzed using SPSS version 22 (SPSS Inc., Chicago, IL, USA) and will be considered statistically significant at a *p* value < 0.05.

## Discussion

The CRONuS trial presents clinic relevance and is necessary since studies have revealed that the conventional rehabilitation undergone by patients after stroke improves their functional independence. It is expected that cardiovascular rehabilitation will improve the structural and functional cardiac alterations—as assessed by echocardiography and the biochemical profile—of patients in the intervention group, and that the amelioration of these parameters after the cardiovascular rehabilitation will have a favorable impact on functional capacity, as shown by improvements in the 6-min walk test distance, VO_2max_, and quality of life in patients after stroke.

Cardiac rehabilitation is a widely used protocol in older people with cardiovascular disease. While physical exercise is well accepted for secondary prevention of cardiovascular disease after stroke, it is also indicated for primary prevention. Our findings may be applicable towards improving the practice of cardiac rehabilitation of patients in the chronic phase of ischemic stroke to improve cardiovascular outcome and reduce its recurrence (Additional file 1).

## Trial status

This trial is ongoing.

## Additional file

Additional file 1: SPIRIT 2013 checklist: Recommended items to address in a clinical trial protocol and related documents. (DOCX 52 kb)

## Abbreviations

ADL: Activities of daily living
CG: Control group
DM: Diabetes mellitus
EuroQol: The European (5D) Quality of Life Scale
IG: Intervention group
IVSDT: Interventricular septum diastolic thickness
LAV: Left atrial volume
LVDD: Left ventricular diastolic diameter
LVEF: Left ventricular ejection fraction
LVM: Left ventricular mass
LVRT: Left ventricular relative thickness
LVSD: Left ventricular systolic diameter
mRS: Modified Rankin Scale
NIHSS: National Institutes of Health Stroke Scale
PWDT: Posterior wall diastolic thickness
THR: Target heart rate
VO_2_ max: Maximum oxygen capacity
